# Case Report: Nintedanib for Pembrolizumab-Related Pneumonitis in a Patient With Non-Small Cell Lung Cancer

**DOI:** 10.3389/fonc.2021.673877

**Published:** 2021-06-18

**Authors:** Xiao-Hong Xie, Hai-Yi Deng, Xin-Qing Lin, Jian-Hui Wu, Ming Liu, Zhan-Hong Xie, Yin-Yin Qin, Cheng-Zhi Zhou

**Affiliations:** State Key Laboratory of Respiratory Disease, National Clinical Research Centre for Respiratory Disease, Guangzhou Institute of Respiratory Health, The First Affiliated Hospital of Guangzhou Medical University, Guangzhou Medical University, Guangzhou, China

**Keywords:** checkpoint inhibitor-related pneumonitis, nintedanib, steroid therapy, non-small cell lung cancer, pembrolizumab

## Abstract

Pembrolizumab, an immune checkpoint inhibitor (ICI) approved for advanced non-small cell lung cancer (NSCLC) treatment, has shown superior survival benefits. However, pembrolizumab may lead to severe immune-related adverse events (irAEs), such as checkpoint inhibitor-related pneumonitis (CIP). The routine treatment of CIP was based on systemic corticosteroids, but the therapies are limited for patients who are unsuitable for steroid therapy. Here, we present the first successful treatment of nintedanib for pembrolizumab-related pneumonitis in a patient with advanced NSCLC.

## Introduction

Introduction of immune checkpoint inhibitor (ICI) therapy leads to a significant survival improvement in various tumors (1). Pembrolizumab has been found to be superior to other chemotherapeutic agents as first-line treatment in metastatic non-small cell lung cancer (NSCLC) (2). However, pembrolizumab may lead to severe immune-related adverse events (irAEs), such as checkpoint inhibitor-related pneumonitis (CIP) (3). The real-world incidence of CIP in patients with NSCLC is reported up to 19% for all grades (4), and the routine treatment of CIP is based on systemic corticosteroids. However, the optional therapies are limited if patients are not sensitive to steroid therapy.

Nintedanib, an oral tyrosine kinase inhibitor, has been approved for slowing progression of idiopathic pulmonary fibrosis (IPF) (5). Nintedanib blocks the vascular endothelial growth factor (VEGF), the platelet-derived growth factor receptor (PDGF) and the fibroblast growth factor receptor (FGFR). All these receptors are involved in cancer development, therefore nintedanib is considered to possess an antitumor effect (6). For instance, nintedanib in combination with docetaxel acts as the second-line therapy for advanced NSCLC (7). Nintedanib plus paclitaxel may improve PD-L1 treatment for metastatic triple negative breast cancer (8). Two studies have given clues that nintedanib may prevent anticancer therapy-related pneumonitis. Fang et al. found that nintedanib has a dramatic effect on targeted therapy-related interstitial pneumonia, providing a promising strategy for the patients who are not suitable for corticosteroid therapy (9). Unlike targeted therapy-associated pneumonitis, CIP may be caused by excessive autoimmune response of tumor infiltrating lymphocytes (10). Interestingly, Yamakawa and colleagues reported that the addition of nintedanib to prednisolone prevented atezolizumab-induced pneumonitis in IPF combined with NSCLC (11). It is worth noting that as an immunosuppressor, VEGF may function by inhibiting dendric cells (DCs) maturation and immigration, along with promoting PD-L1 expression by DCs. Since nintedanib can target the VEGF pathway, we assume it may prevent ICI-induced pneumonitis. Here, we present a successful treatment of nintedanib for pembrolizumab-related pneumonitis in a patient with advanced NSCLC.

## Case Presentation

**Figure 1:**
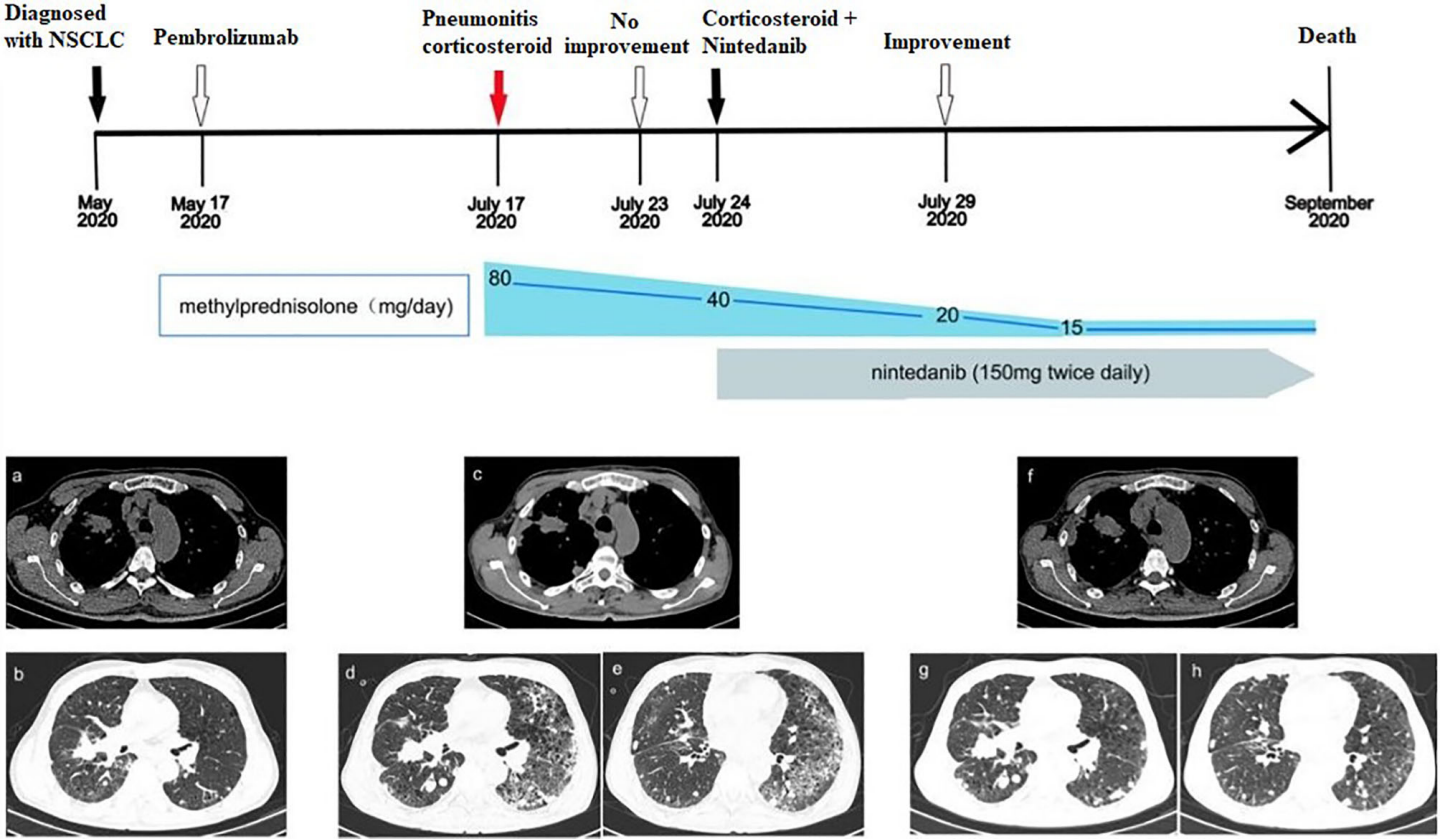
Development of ICI-related pneumonitis in a patient treated with pembrolizumab for lung adenocarcinoma. **(A, B)** Chest CT images showed the lesions in the upper lobe of the right lung before treatment (May 17, 2020). **(C–E)** After 3 cycles of immunotherapy, CT images showed that primary lesions were stabilized, but pneumonitis in the left lung (July 24, 2020). **(F–H)** CT images showed obvious improvement of pneumonitis (July 29, 2020).

A 58-year-old man was sent to a local hospital with shortness of breath. He denied any history of chronic pulmonary diseases. However, chest computed tomography (CT) scan in May 2020 showed a mass in the upper lobe of the right lung (**Figures 1A, B**). The patient was diagnosed with lung adenocarcinoma with bone metastasis after a thorough work-up, and the pathological examination revealed it to be driver gene-negative. He strongly refused chemotherapy and insisted to receive immunotherapy. He was then administered 100 mg of pembrolizumab (2 mg/kg, every 3 weeks) combined with bevacizumab by the local physician on May 17, 2020. After the third pembrolizumab infusion, he complained of fever (38.4°C) and shortness of breath. Pembrolizumab was discontinued and oxygen inhalation was required. After receiving methylprednisolone (80 mg/day) for a week, he reported no significant symptom alleviation. Therefore, he was transferred immediately to our hospital. On admission, he presented with acute dyspnea. His blood pressure was normal, but his blood oxygen saturation was 96%. Laboratory tests revealed the serum tumor markers were within the normal range except for elevated Krebs von den Lungen-6 (KL-6) (1575 U/mL), and no abnormalities in white blood cell count, lactate dehydrogenase and C-reactive protein. A repeat chest CT scan showed multiple patchy and striped shadows in the left lung on July 24 (**Figures 1C–E**), and stable disease (RECIST criteria) of primary tumor lesion was evaluated. Pulmonary function tests indicated restrictive ventilatory defect. Microbiological testing of bronchoalveolar lavage fluid were negative (including staining and culture for bacteria, fungi, viruses, mycobacteria). The diagnosis of grade 3 pembrolizumab-related pneumonitis was based on the above findings. The initial dosage of methylprednisolone (80mg/day) did not improve his clinical symptoms and we decided to deliver him with methylprednisolone (40 mg/day) and added nintedanib (150 mg bid) to CIP treatment. Five days later, his clinical condition greatly improved, with obvious radiological improvement in the left lung and stable primary tumor lesion on follow-up CT (**Figures 1F–H**). Since we were concerned about the side effects of corticosteroids, we tried to lower the dose of methylprednisolone to 20 mg/day, but nintedanib (150 mg bid) was continued. After 2 weeks of nintedanib monotherapy, the patient’s condition improved so he withdrew nintedanib. Unfortunately, he experienced rapid metastatic progression and died 2 months later.

## Discussion

The patient developed CIP after the third injection of pembrolizumab. According to radiographic classification, this case is interstitial pneumonia (12). Laboratory test showed that his serum KL-6 was markedly increased. KL-6 is regarded as an important biomarker for interstitial lung disease (ILD) and classified as MUC1 mucin protein, and regenerating type II pneumocytes are the primary cellular source of KL-6/MUC1 in the affected lungs of patients with ILD. Serum KL-6/MUC1 is elevated in 70% to 100% of patients with various ILDs such as idiopathic interstitial pneumonias, radiation pneumonitis, drug-induced ILD and acute respiratory distress syndrome (13).

Clinicians should be fully aware of the possibility of ICI-induced pulmonary toxicity when using programmed death-1/programmed death-ligand 1 (PD-1/PD-L1) inhibitors. CIP is one of the most common fatal adverse events of PD-1/PD-L1 inhibitors (14). It has been reported that approximately 70% to 80% of CIP patients can be controlled by routine corticosteroid treatment (15). One-week treatment of initial dosage of methylprednisolone (80mg/day) did not improve the patient’s clinical symptoms, indicating that CIP had a poor response to high-dose of methylprednisolone. The patient was not sensitive to single agent corticosteroid, and thus the course of pulmonary fibrosis was not reversed. After that, we attempted to add nintedanib to treat CIP. Thus, methylprednisolone (40 mg/day) plus nintedanib (150 mg bid) was administered. Unexpectedly, the improved general situation of the patient and the remitted clinal symptoms within 5 days indicated that pembrolizumab-associated pneumonitis had a favorable response to the combination therapy of methylprednisolone and nintedanib. We decreased the dose of methylprednisolone by half dose because we think the dosage of corticosteroid should be reduced after treatment has achieved remission of symptoms. The addition of nintedanib to CIP treatment may reduce the dosage and even duration use of corticosteroid. In some practice, immune suppressants are recommended for CIP treatment (4, 15), but we did not try immunosuppressors such as mycophenolate mofetil and cyclophosphamide for CIP treatment because they work slowly, which limits their use in CIP because of its acute or subacute course.

Although there have never been trials of combination treatment of ICIs and nintedanib until now, this combination therapy may prevent and treat CIP. To the best of our knowledge, this is the first report to provide clinical evidence for nintedanib in the treatment of earlier onset of ICI-induced CIP. One possible mechanism is nintedanib inhibits pulmonary fibrosis by targeting VEGFR (16). Alternatively, nintedanib may reduce lung exudation and promote lung recovery through suppressing VEGF (17). The patient was administered pembrolizumab plus bevacizumab as first-line treatment for NSCLC. The anti-VEGF effect of nintedanib can only partially explain its prophylaxis for refractory CIP because bevacizumab (an anti VEGF antibody) does not have this effect on CIP. Besides VEGFR family, nintedanib targets pro-angiogenic and pro-fibrotic pathways mediated by PDGFR, FGFR and as well as Src and Flt-3 kinases, which are related to the pathogenesis of pulmonary fibrosis. Nintedanib competitively binds to the ATP of these receptors, blocking the proliferation, migration, and signaling transduction of fibroblasts, which might prevent CIP.

VEGF inhibition can enhance immunotherapy benefit for a variety of cancers (18). We think that the local physician had the hypothesis that concurrent VEGF blockade may enhance the anti-tumor activity of pembrolizumab for NSCLC patients. However, stable disease of primary tumor lesion was evaluated on his chest CT after three cycles of pembrolizumab plus bevacizumab. During CIP, pembrolizumab was stopped, but nintedanib was continued. If nintedanib and PD1 immunotherapy would be administered to the patient with NSCLC, will the combination therapy have both anticancer efficacy and prophylaxis for CIP? At the moment, we have insufficient experience in the use of nintedanib, but it seems that nintedanib should be used at an earlier onset of CIP.

In conclusion, nintedanib combined with corticosteroid therapy might be an option for patients with CIP, especially for those with poor response to steroid-based therapy. Further studies are urgently needed to elucidate the detailed mechanism of combined regimen strategy to prevent and treat CIP.

## Data Availability Statement

The original contributions presented in the study are included in the article/supplementary Material. Further inquiries can be directed to the corresponding author.

## Ethics Statement

Written informed consent was obtained from the individual(s) for the publication of any potentially identifiable images or data included in this article.

## Author Contributions

X-HX treated the case and wrote the manuscript. H-YD, X-QL, J-HW, ML, Z-HX, and Y-YQ treated the case. C-ZZ contributed to PET/CT response evaluation. All authors contributed to the article and approved the submitted version.

## Funding

This study was supported by Beijing Xisike Clinical Oncology Research Foundation (Y-XD2019-136, Y-2019Genecast-076).

## Conflict of Interest

The authors declare that the research was conducted in the absence of any commercial or financial relationships that could be construed as a potential conflict of interest.
